# Age at diagnosis, but not HPV type, is strongly associated with clinical course in recurrent respiratory papillomatosis

**DOI:** 10.1371/journal.pone.0216697

**Published:** 2019-06-13

**Authors:** Farrel J. Buchinsky, William L. Valentino, Nicole Ruszkay, Evan Powell, Craig S. Derkay, Riaz Y. Seedat, Virgilijus Uloza, Frederik G. Dikkers, David E. Tunkel, Sukgi S. Choi, Anthony J. Mortelliti, Paolo Campisi, Juan C. Ospina, Adam J. Donne, Robert T. Sataloff, Stephen F. Conley, John E. McClay, Ellen M. Friedman, Lisa Elden, Dale A. Tylor, Clark A. Rosen, Libby J. Smith, Graeme J. Copley, David E. Karas, John M. Schweinfurth, Charles M. Myer, Brian J. Wiatrak, Joseph E. Dohar, Steven E. Sobol, Robert W. Bastian, Richard J. H. Smith, Marshall E. Smith, Abebe M. Wassie, James C. Post, Garth D. Ehrlich

**Affiliations:** 1 Respiratory Papillomatosis Program, Allegheny Health Network, Pittsburgh, Pennsylvania, United States of America; 2 Drexel University College of Medicine, Philadelphia, Pennsylvania, United States of America; 3 Pittsburgh Clinical Genomics Laboratory, UPMC Magee—Women’s Hospital, Pittsburgh, Pennsylvania, United States of America; 4 Department of Otolaryngology—Head and Neck Surgery, Eastern Virginia Medical School, Norfolk, Virginia, United States of America; 5 Department of Otorhinolaryngology, University of the Free State, Bloemfontein, South Africa; 6 Department of Otorhinolaryngology, Lithuanian University of Health Sciences, Kaunas, Lithuania; 7 Department of Otorhinolaryngology, Amsterdam UMC, Amsterdam, Netherlands; 8 Department of Otolaryngology—Head and Neck Surgery, Johns Hopkins Medical Institutions, Baltimore, Maryland, United States of America; 9 Department of Otolaryngology and Communication Enhancement, Harvard Medical School—Boston Children's Hospital, Boston, Massachusetts, United States of America; 10 Department of Otolaryngology and Communication Sciences, State University of New York Upstate Medical University, Syracuse, New York, United States of America; 11 Department of Otolaryngology—Head and Neck Surgery, Hospital for Sick Children, University of Toronto, Toronto, Ontario, Canada; 12 Unit of Otorhinolaryngology and Maxillofacial Surgery, Pontificia Universidad Javeriana-Hospital San Ignacio, Bogotá, Colombia; 13 Department of Paediatric Otolaryngology, Alder Hey Children's NHS Foundation Trust, Liverpool, United Kingdom; 14 Department of Otolaryngology—Head and Neck Surgery, Drexel University College of Medicine, Philadelphia, Pennsylvania, United States of America; 15 Department of Otolaryngology and Communication Sciences, Medical College of Wisconsin, Milwaukee, Wisconsin, United States of America; 16 Frisco ENT for Children, Dallas, Texas, United States of America; 17 Otolaryngology and Pediatrics, Baylor College of Medicine, Houston, Texas, United States of America; 18 Department Otolaryngology at Children's Hospital of Philadelphia, University of Pennsylvania, Philadelphia, Pennsylvania, United States of America; 19 ENT Associates of Santa Barbara, Santa Barbara, California, United States of America; 20 Department of Otolaryngology, University of California San Francisco, San Francisco, California, United States of America; 21 University of Pittsburgh Voice Center, University of Pittsburgh School of Medicine, Pittsburgh, Pennsylvania, United States of America; 22 Red Cross War Memorial Children’s Hospital, Cape Town, South Africa; 23 Section of Otolaryngology, Yale University School of Medicine, New Haven, Connecticut, United States of America; 24 Department of Otolaryngology, University of Mississippi Medical Center, Jackson, Mississippi, United States of America; 25 Department of Otolaryngology–Head and Neck Surgery, Cincinnati Children's Hospital Medical Center, Cincinnati, Ohio, United States of America; 26 Pediatric Otolaryngology, Children's Hospital of Alabama, Birmingham, Alabama, United States of America; 27 Division of Pediatric Otolaryngology, Children's Hospital of Pittsburgh—UPMC, Pittsburgh, Pennsylvania, United States of America; 28 Bastian Voice Institute, Downers Grove, Illinois, United States of America; 29 Division of Pediatric Otolaryngology, University of Iowa Hospitals and Clinics, Iowa City, Iowa, United States of America; 30 Division of Otolaryngology—Head and Neck Surgery, University of Utah School of Medicine, Salt Lake City, Utah, United States of America; 31 Department of Otolaryngology—Head and Neck Surgery, Addis Ababa University, Addis Ababa, Ethiopia; 32 Departments of Microbiology and Immunology, and Otolaryngology—Head and Neck Surgery and Center for Genomic Sciences, Institute of Molecular Medicine and Infectious Disease, Drexel University College of Medicine, Philadelphia, Pennsylvania, United States of America; Federal University of Sergipe, BRAZIL

## Abstract

**Background:**

Recurrent Respiratory Papillomatosis (RRP) is a rare disease characterized by the growth of papillomas in the airway and especially the larynx. The clinical course is highly variable among individuals and there is poor understanding of the factors that drive an aggressive vs an indolent course.

**Methods:**

A convenience cohort of 339 affected subjects with papillomas positive for only HPV6 or HPV11 and clinical course data available for 1 year or more, from a large multicenter international study were included. Exploratory data analysis was conducted followed by inferential analyses with frequentist and Bayesian statistics.

**Results:**

We examined 339 subjects: 82% were diagnosed prior to the age of 18 years, 65% were infected with HPV6, and 69% had an aggressive clinical course. When comparing age at diagnosis with clinical course, the probability of aggressiveness is high for children under five years of age then drops rapidly. For patients diagnosed after the age of 10 years, an indolent course is more common. After accounting for confounding between HPV11 and young age, HPV type was minimally associated with aggressiveness. Fast and Frugal Trees (FFTs) were utilized to determine which algorithms yield the highest accuracy to classify patients as having an indolent or aggressive clinical course and consistently created a branch for diagnostic age at ~5 years old. There was no reliable strong association between clinical course and socioeconomic or parental factors.

**Conclusion:**

In the largest cohort of its type, we have identified a critical age at diagnosis which demarcates a more aggressive from less aggressive clinical course.

## Introduction

Recurrent Respiratory Papillomatosis (RRP) is a rare disorder characterized by the development of papillomas within the respiratory tract. It is caused by HPV6 and 11 (and rarely HPV16, 18 and 45); its transmission is presumed to occur as the fetus passes through an infected birth canal. For adults, the leading theory for the transmission of HPV is inoculation during sexual activity, although latency for several decades from acquisition at birth is possible.

The clinical course of RRP is highly variable [1]. Symptoms can range from mild dysphonia to acute respiratory distress. The disease may undergo spontaneous remission or require multiple surgeries per year to maintain airway patency. The age at onset is also highly variable, and ranges from the neonate to the elderly [2], with terms such as juvenile-onset RRP (JoRRP) and adult-onset RRP (AoRRP) often used to reflect these differences. Notwithstanding the different terms, there is no histological distinction between them. Most publications focus on JoRRP exclusively or patients who are exclusively adults, but herein we analyze a sample of patients that range from neonates to geriatrics.

The clear majority of previous studies have concluded that HPV11 is associated with a more aggressive disease course as compared to HPV6. Although clinicians have wondered whether knowledge of HPV type would prognosticate clinical course, this concept has been challenged by some authors who have demonstrated that age of onset is a better indicator of clinical course than HPV type [3–5].

The data presented herein are from the largest collection of RRP patients for whom HPV type is known. The number of unique affected subjects is 3 times greater than we have previously presented; includes subjects from many countries; and is no longer limited to JoRRP. Using this expanded data set, we include subjects across the entire age spectrum to delineate variables associated with a more aggressive course of disease (defined in “statistical analysis”). The data should not be conflated with an epidemiologic study describing the incidence, demographics and clinical course or RRP. The study is not a random worldwide sampling of people with RRP. As such, this sample is useful for internal comparisons, but its frequencies are unlikely to be generalized to *all* persons affected with RRP.

## Materials and methods

### Sample composition

From 2003 through 2014, a total of 611 unique affected subjects were enrolled with informed consent in an NIH-funded multicenter study “Genetic Susceptibility to Papilloma-induced Voice Disturbance.” The coordinating site was located at Allegheny General Hospital, Pittsburgh, PA [6]. The study was approved as the coordinating center by the Institutional Review Board (IRB) of the Allegheny-Singer Research Institute under research protocol number 3145. Written Informed consent was documented for every subject. Each of the satellite centers obtained approval through their own IRB and all subjects (or their parents in the case of minors) provided written documented informed consent.

Attending otolaryngologists at 35 centers in 8 countries (23 in the United States) enrolled subjects. Subjects underwent debridement procedures for clinical purposes and not for research purposes. During such clinical procedures, the otolaryngologist obtained two biopsies which were submitted together with the clinical data to the coordinating site. Metrics in the clinical data included: the total number of airway procedures (count); the frequency of procedures (count per year); the days since the last papilloma-reduction procedure; involvement in the trachea or distally (binomial). The parents of children affected with RRP or the patients themselves (if above the age of 18) filled out a survey that included questions related to additional HPV burden in the family, maternal educational status and gross household income.

Beginning with 611 subjects, a subset of 339 patients with a fresh papilloma specimen that was positive for either HPV6 or HPV11(but not both) and who had at least 1 year or more of post-diagnosis clinical course data were included in this study. We excluded the few (n = 8) patients with more than one HPV type since we did not know if it was the HPV6 or the HPV11 or both that was driving the clinical phenotype. Other reasons for exclusion were: no fresh biopsy specimen (since many patients were enrolled through a patient support group and not through their surgeon); patients who were in remission following enrollment (n = 157); patients with insufficient follow up since diagnosis < 0.9 years (n = 98); patients whose HPV was not typed (n = 24); patients who had HPV types other than HPV6 or HPV11 (n = 14); patients with coinfection of HPV6 and HPV11 (n = 12); a patient with coinfection of HPV11 and HPV16; a patient with HPV18; patients with incomplete clinical data to determine composite aggressiveness (n = 13). Many of the excluded patients met multiple exclusion criteria.

### Specimen collection and DNA extraction

During surgery to control the patient’s disease, two 1 mm^3^ papilloma biopsies were collected and preserved in TRIzol. At the coordinating center, DNA was extracted from the laryngeal biopsies according to the manufacturer’s guidelines (Invitrogen, Carlsbad, CA, USA). To assess the quantity and quality of the isolated DNA, samples were tested by spectrophotometry using a ND-1000 spectrometer (NanoDrop) and by PCR using the GH20 (59-GAAGAGCCAAGGACAGGTAC-39) and PC04 (59-CAACTTCATCCACGTTCACC-39) primers, which span a 268-bp segment of the β-globin gene [7]. During the early phase of establishing the specimen collection, several samples failed to yield β-globin amplicons. Thus, the specimens that failed PCR were subjected to whole genome amplification (WGA) using the GenomiPhi DNA Amplification Kit from Amersham Biosciences (Piscataway, NJ, USA) following the manufacturer’s instructions. After the extraction of the first 45 laryngeal specimens, we decided to systematically carry out WGA on all extracted DNA. The products of the WGA reactions were used as template to PCR amplify the β-globin gene using GH20 and PC04 primers.

### HPV typing

In the initial years, typing was accomplished using Type-Specific PCR primers (TS-PCR) and Restriction Fragment Length Polymorphisms (RFLP) [3]. In the later 2000s, PCR amplification using the consensus primers PGMY09/11 was used [8]. The resulting ~450 base pair amplimers were confirmed by agarose gel electrophoresis and Sanger sequencing of the L1 sequence and then blasting the derived sequence against the GENBANK reference sequences of HPV types 6, 11, 16, 18 and 45. More recently, the Linear Array HPV Genotyping Test Kit (Roche Molecular Systems, Inc., Pleasanton, CA, USA) was used. Many samples were typed by two different methods for quality control.

### Statistical analyses

Prior to subject enrollment, the cutoff value in differentiating adult-onset and juvenile-onset-RRP (AoRRP and JoRRP, respectively) was defined as 18 years of age and an aggressive clinical course was defined as a composite phenotype where any one (or more) of the following four criteria were met: more than or equal to 10 total surgeries, more than or equal to four surgeries per year, distal spread to the trachea and/or beyond, or requiring a tracheotomy at any stage. These criteria were first declared by Doyle et al [9] and have been referred to and used in many other papers [3,5,10–12]. Other researchers have preferred the term “severe” and contrast that state to “mild/moderate” and have used similar but different measures and criteria in labeling the disease course (discussed below). Composite phenotypes are frequently used as endpoints or outcomes in clinical trials; they are characterized by combining multiple features for statistical efficiency. Among those surgeons managing RRP, there is an intuitive appreciation for what constitutes an aggressive case. Yet, two different individuals could be aggressive for different reasons (e.g. tracheotomy vs many surgeries). The statistical models in this paper use composite aggressiveness; and, for reading simplicity, we simply use the term “aggressive”.

Latency was defined as time from birth to time of diagnosis with JoRRP; and in AoRRP, it was defined as time of sexual debut to time of diagnosis. While clinical course was considered a dependent outcome measure (aggressive vs indolent) the following variables were considered to be independent potential predictor variables: HPV type, age at diagnosis, age at enrollment, gender, race, ethnicity (Hispanic vs. non-Hispanic) educational status of mother, gross household income, parental genital warts, route of delivery (vaginal vs cesarean), birth order, maternal sexual debut, affected individual sexual debut, other RRP in family. Gender, race and ethnicity were provided by the surgeon and were presumably ascertained by a combination of the surgeon’s assumption from all that he or she knew from interacting with the subject and their family. Race and ethnicity categories were selected to comply with the reporting requirements of the National Institute on Deafness and Other Communication Disorders (NIDCD).

The dataset was initially inspected by conducting a descriptive statistical summary of all variables in isolation (R statistical software (http://www.r-project.org/)) and inspecting boxplots and histograms of each variable. Variables that did not follow a Gaussian distribution were transformed by taking the natural logarithm of the value (age at diagnosis, age at enrollment, total number of procedures, frequency of procedures per year, time from last operation to time of current operation and gross household income). Initial analyses consisted of a Fisher exact test of independence for binomial variables: HPV type (6 vs 11) and clinical course (aggressive vs indolent). Multiple logistic regression was then used to analyze independent variables while controlling for multiple other variables in the model. A series of models was used to analyze the most recent intersurgical interval (log number of days) with multiple linear regression.

We used both frequentist and Bayesian statistics. Frequentist statistics applies the commonly used null hypothesis significance testing utilizing p-values. In doing so, it essentially works backwards by calculating the probability of our collected data if the null hypothesis were to be true. Bayesian statistics works forward by calculating the probability of the hypothesis given the data [13]. Bayesian output is less susceptible to confident misunderstanding and misuse as is common with p-values [14]. No adjustments have been made for multiple testing. The data set is large by RRP standards, and thus the traditional frequentist statistics cutoff at p = 0.05 should be viewed with caution.

Finally, associations were explored with Fast and Frugal Trees (FFTs) [15]. FFTs are essentially a tool for classification used as decision trees to predict a complex set of data in a fast, easy and cognitively simple way. The final tree may be of use, but it also serves to demonstrate which factors are most important in differentiating outcomes and at what level to set any cutoff.

## Results

We evaluated 339 subjects, most had JoRRP, most were infected with HPV6, and most had run an aggressive clinical course (Table 1). 233 subjects met the composite criteria to be labelled “aggressive”. The most frequent component was surgical count ≥ 10 (192/233), followed by maximum frequency in 12 months ≥ 4 (169/233). Recall that this data set is not suitable for describing the proportion of all RRP that is aggressive. We believe that this convenience cohort is enriched for those with aggressive course.

**Table 1:** Characteristics of a convenience cohort of patients with RRP by Composite Aggressive vs. Indolent Clinical Course.

	All	Indolent	Aggressive	P-value
	N = 339	N = 106	N = 233	
	Mean + (SD) or Median + [IQR] or Count + (%)	Mean + (SD) or Median + [IQR] or Count + (%)	Mean + (SD) or Median + [IQR] or Count + (%)	
**Surgical Count**	28 (49)	4.3 (2.4)	38 (56)	
			*82% ≥ 10*	
**Max Frequency** (count per year)	5.0 (5.3)	1.7 (0.8)	6.5 (5.8)	
			*73% ≥ 4*	
**Tracheotomy**	41 (12%)	0 (0%)	41 (18%)	
**Distal Spread**	73 (22%)	0 (0%)	73 (31%)	
**Last Surgical Interval**^a^ (days)	120 [60;330]	300 [150;913]	90 [47;196]	<0.001^b^
**Age at Onset**				<0.001^c^
Adult	61 (18%)	43 (41%)	18 (7.7%)	
Juvenile	278 (82%)	63 (59%)	215 (92%)	
**Age at Diagnosis** (years)	3.9 [1.8;8.3]	10 [4.0;33]	2.9 [1.5;5.0]	<0.001^b^
**Trichotomous Age at Diagnosis**				<0.001^c^
0 to 4.9 years	207 (61%)	32 (30%)	174 (75%)	
5.0 to 9.9 years	56 (17%)	20 (19%)	36 (16%)	
≥ 10 years	76 (22%)	54 (51%)	22 (9.4%)	
**Follow-Up** (years)	4.1 [2.0; 9.5]	3.2 [1.7; 7.3]	5.3 [2.2; 10.8]	0.004
**HPV type**				0.014 ^c^
6	219 (65%)	79 (75%)	140 (60%)	
11	120 (35%)	27 (26%)	93 (40%)	
**Sex**				0.500 ^c^
Female	146 (43%)	49 (46%)	97 (42%)	
Male	193 (57%)	57 (54%)	136 (58%)	
**Ethnicity**				0.156 ^d^
Hispanic	28 (8.3%)	5 (4.7%)	23 (9.9%)	
Non-Hispanic	303 (89%)	97 (92%)	206 (89%)	
Not Recorded	8 (2.4%)	4 (3.8%)	4 (1.7%)	
**Race**				0.917 ^d^
Asian	3 (0.9%)	1 (0.9%)	2 (0.9%)	
Black	108 (32%)	31 (29%)	77 (33%)	
Not Recorded	2 (0.6%)	0 (0%)	2 (0.9%)	
Other	16 (4.7%)	5 (4.7%)	11 (4.7%)	
White	210 (62%)	69 (65%)	141 (61%)	
**Country**				<0.001^d^
Canada	23 (6.8%)	10 (9.4%)	13 (5.6%)	
Columbia	6 (1.8%)	2 (1.9%)	4 (1.7%)	
Ethiopia	6 (1.8%)	3 (2.8%)	3 (0.4%)	
Lithuania	32 (9.4%)	23 (22%)	9 (3.9%)	
Netherlands	6 (1.8%)	1 (0.9%)	5 (2.2%)	
South Africa	51 (15%)	14 (13%)	37 (16%)	
United Kingdom	2 (0.6%)	1 (0.9%)	1 (0.4%)	
USA	213 (63%)	52 (49%)	161 (69%)	

For some variables (closer to a normal distribution) the mean and SD are shown; for other variables, median and IQR are shown. Dichotomous and categorical variables were simply counted. SD = Standard Deviation. IQR = Interquartile Range.

^a^ Surgical Interval is defined as the number of days between the previous debridement and the one that took place on the day of enrollment. The day of enrollment is when the biopsy of their papilloma was taken.

^b^ Wilcoxon signed-rank test.

^c^ Chi-Squared Test.

^d^ Fisher-Exact Test.

### HPV11 is associated with aggressive disease; younger age at diagnosis is also associated with aggressive disease

The dependent variable is the disease course (aggressive vs indolent). If one analyzes just one independent variable at a time (univariate analysis) then the results herein recapitulate previous research [12,16] demonstrating that HPV11 is associated with a more aggressive clinical course (chi-squared test—see HPV row of Table 1; logistic regression—see By HPV alone model of Table 2). Also, in keeping with previous data sets, this study shows that younger age at diagnosis is associated with an aggressive clinical course. Table 1 shows that 92% of those with an aggressive course had juvenile onset but only 59% of those with an indolent course had juvenile onset; the median age at diagnosis is 7.4 years younger for those with an aggressive course. Table 2 shows multiple ways of modeling the age at diagnosis as the only independent variable with clinical course as the outcome variable using logistic regression (by JoRRP vs AoRRP alone, by age at diagnosis alone). The odds of JoRRP being aggressive is 8 times that of AoRRP being aggressive, while the odds of HPV11 being aggressive is only 2 times that of HPV6 being aggressive. Furthermore, the Akaiki information criterion (AIC) score is much lower for age at diagnosis, indicating a better model fit.

**Table 2:** Multiple logistic regression models in which composite aggressiveness is a function of age at diagnosis and HPV type.

Model of Aggressiveness	Metric	P-value	Odds Ratio	AIC
By HPV type alone	HPV11 vs HPV6	0.011	1.94	418
By onset alone	Juvenile vs Adult	< 0.001	8.15	376
By age at diagnosis alone	ln(age)	< 0.001	0.46*	365
By HPV type AND onset	Juvenile vs Adult	< 0.001	7.68	375
	HPV11 vs HPV6	0.084	1.62	
By HPV type AND age at diagnosis	ln(age)	< 0.001	0.47	367
	HPV11 vs HPV6	0.413	1.26	
By HPV type AND onset AND allowing for interaction	Juvenile vs Adult	< 0.001	5.28	372
	HPV11 vs HPV6	0.221	0.36	
	Juvenile:HPV11	0.045	5.93	
By trichotomous age at diagnosis alone	Age 5–10 vs Age > 10	< 0.001	4.42	349
	Age 0–5 vs Age > 10	< 0.001	13.42	
By trichotomous age AND HPV	Age 5–10 vs Age > 10	< 0.001	4.43	350
	Age 0–5 vs Age > 10	< 0.001	12.71	
	HPV11 vs HPV6	0.288	1.37	

AIC refers to the Akaike information criterion; the lower the number is, the better the model fits the data. The AIC “penalizes” the score for adding additional variables or levels to prevent overfitting.

*The odds of being aggressive given that one was diagnosed at any particular age are 0.46 of the odds if one was diagnosed at that age times Euler's number (2.72 since we used the natural logarithm (ln), e.g. age 2.0 years vs 5.4 years or 7 vs 19 years).

### HPV11 is associated with a younger age at diagnosis

The age at diagnosis is lower in those infected with HPV11 (as demonstrated by 3 different statistical methods: Student's t-test of logarithmically transformed age at diagnosis, Wilcoxon rank sum, Bayesian estimation supersedes the t test (BEST)). Among JoRRP, the Bayesian estimate mean age at diagnosis was 1.34 years less for HPV11 versus HPV6 (2.29 years vs 3.63 years; 95% high density interval for difference, 0.75 to 1.91 years). Cohen’s d showed a medium-large effect size (0.67), and 0.0% of the simulations showed HPV6 with an equal or lower mean age at diagnosis. Among AoRRP, the mean age at diagnosis for HPV11 was 6.8 years less than HPV6 (38 vs 31 years, 95% HDI for difference was 0.020 to 13.7 years). Cohen’s d showed a medium effect size (0.60), but 2.7% of the posterior probability estimates revealed that the mean age at diagnosis was lower for HPV6 than 11. By traditional frequentist statistics, these AoRRP differences reflect a trend rather than a statistically significant difference. With Bayesian statistics, we can state the following: in JoRRP, there is a 75% probability that the mean age of HPV6-infected subjects is 1.1 or more years older than the mean age of HPV11-infected subjects; in AoRRP the 75% probability difference is 4.5 years or more.

### After controlling for age at diagnosis, the association of HPV11 with aggressiveness is weak and variable

**Fig 1:**
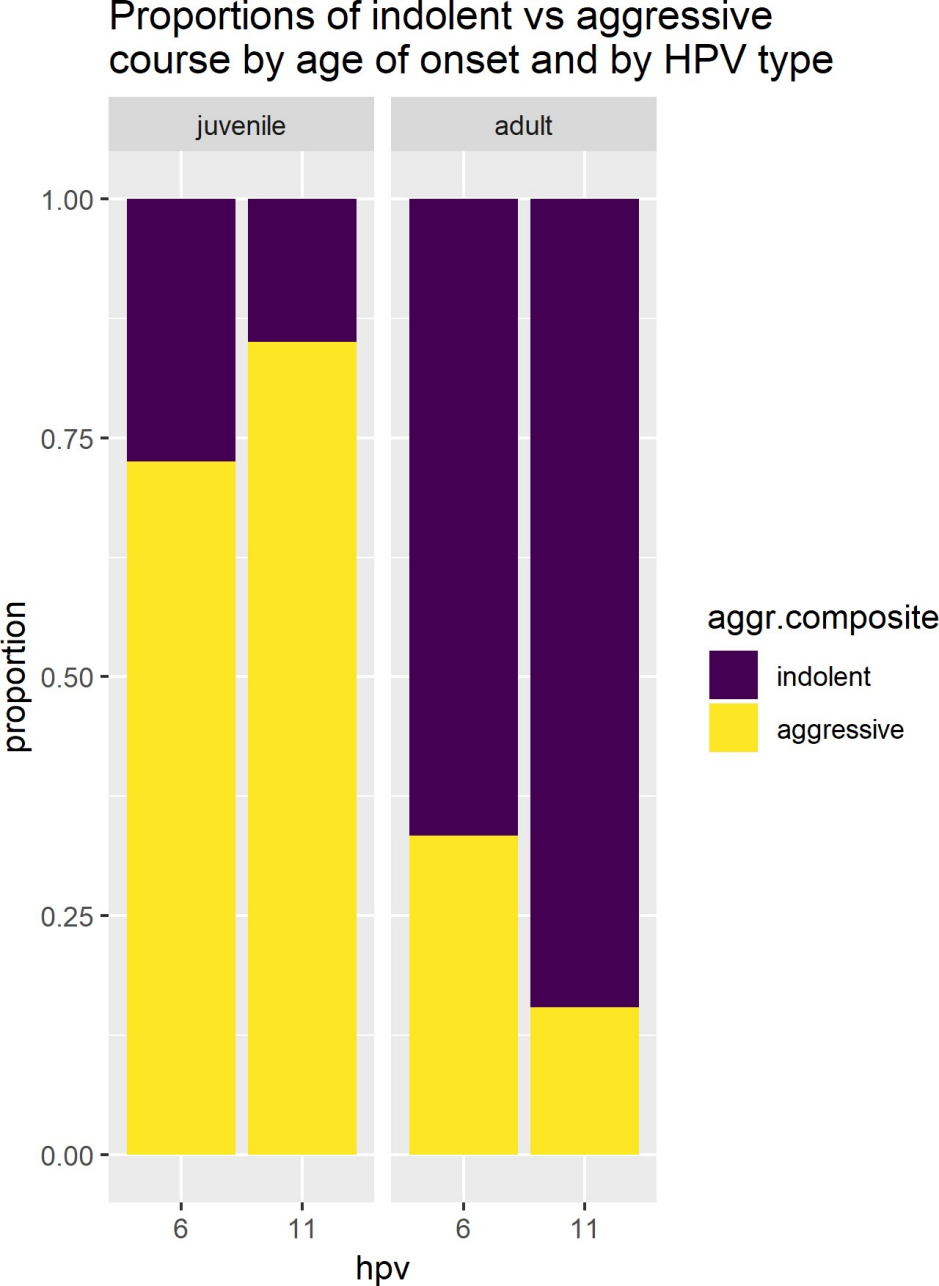
Relationship between clinical course and type of HPV in patients across the spectrum of age at diagnosis.

In multiple logistic regression we are able to deal with confounding variables. We are no longer limited to just one independent variable at a time; instead we can look at both 1) age at diagnosis and 2) HPV type, at the same time while controlling for each other. In multiple logistic regression that controls for age at diagnosis, the p-value for HPV type is statistically insignificant and the AIC score barely changes when we add HPV type over and above age at diagnosis. Across the two HPV types, 73% to 85% of subjects with JoRRP are classified as aggressive but among AoRRP, across the two HPV types, only 15% to 33% of subjects follow an aggressive course (Fig 1). The difference between JoRRP and AoRRP in aggressiveness eclipses the differences associated with HPV type, especially when HPV11 is nested within JoRRP; JoRRP was associated with a statistically significant higher proportion of HPV11 (38%) than in AoRRP (21%). Further weakening any main effect of the HPV type as a factor, in JoRRP the proportion with an aggressive clinical course is higher in those with HPV11, in contrast to AoRRP where the proportion is lower. Multiple logistic regression with an interaction term (see Table 2 “By HPV AND onset AND allowing for interaction”) shows that the way HPV11 is associated with more aggressiveness in JoRRP is statistically significantly different than the way HPV11 is associated with less aggressiveness in AoRRP (p = 0.045). On balance across all ages of onset, an independent effect of HPV type on the composite aggressiveness outcome is not seen once juvenile vs adult onset is considered.

### Clinical course associated with age at diagnosis in three distinct zones

**Fig 2:**
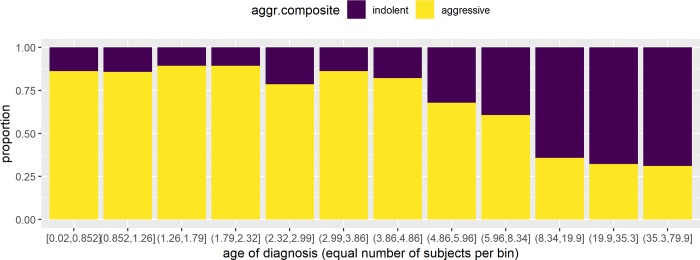
The relationship between age at diagnosis and clinical course. Proportion of patients with an indolent versus aggressive clinical course in relation to the age at which each patient was diagnosed with RRP. Each bar represents one twelfth of the subjects (duo-decile) ranked by age at diagnosis.

Given the central and overwhelming association of age at diagnosis, rather than HPV type, with the disease course, further exploratory data analyses were undertaken. Ignoring HPV type, we ranked all 339 subjects from youngest to oldest at age at diagnosis. A bar plot (Fig 2) shows that the proportion with aggressive disease is high (~80%) for those diagnosed between 0 and 5 years and low (~30%) for those diagnosed at 10 years or older. Between 5 and 10 (n = 56), the proportion with aggressive clinical course is ~60%. Using a manual iterative process, our data set was best modeled with 3 diagnostic age categories (see “By trichotomous age at diagnosis alone” Table 2) with the cutoffs being age 5 years and 10 years. Within each trichotomous category, there was no significant association with age at diagnosis. In other words, age at diagnosis at 2 years old did not seem to be very different from 4 years old. Similarly, age 20 did not seem to be very different from age 40 at diagnosis in terms of proportion who ran an aggressive clinical course. As seen before, when controlling for age at diagnosis, HPV type was not associated significantly with clinical course (see “By trichotomous age AND HPV” in Table 2).

### Clinical course is associated with country and perhaps with maternal condylomata

With trichotomous age of onset identified as the variable with the strongest clinical course association, we systematically added variables to the model. We screened for models that lowered the AIC substantially and whose metric had a p-value less than 0.05. The AIC for multiple logistic regression with trichotomous age and country (controlling for both), was 341; compared to the USA, subjects in Ethiopia (adjusted odds ratio = 0.13, p-value = 0.015), Lithuania (OR = 0.28, p = 0.011) and Canada (OR = 0.29, p = 0.013) had lower odds of aggressiveness. With that age + country model in place, we sought to add additional terms, however none was statistically significant: gender, race, ethnicity, HPV type, other RRP in family, education (of mother for JoRRP, of subject for AoRRP), gross household income, maternal pap smear, cesarean section, paternal condyloma, latency, age of mother at time of birth, time from maternal debut to birth of affected subject.

A history of maternal condyloma did show a trend towards more aggressive clinical disease. If one included only those subjects who answered “yes” or “no” to the survey question (in other words, persons who were unsure were excluded), among 230 remaining subjects, the adjusted odds of an aggressive course were 2.2 times higher if there was a maternal history of condyloma acuminata (p = 0.060, indicating a trend rather than statistical significance). The number of subjects whose mother had had a history of condylomata acuminata was 77, but of those only 49 indicated that they had had the condylomata before the birth of the affected subject and 21 responded that the condylomata had not been present before the birth. The proportion who were aggressive was similar in those whose mother had had condyloma before versus after the birth of the affected individual.

Concerning maternal factors, the well documented triad associated with increased incidence of RRP (namely, young mother (< 20 years old), first born child, vaginal delivery) was explored in our data set where we had the data to do so. There was no association between the complete triad and the disease course. Recall that our study design does not allow us to confirm or refute the relationship with absolute disease incidence. Incidentally, as others have found [17], the complete triad was disproportionately present in JoRRP compared to AoRRP (32% vs 0%, Fisher exact p = 0.007).

Limiting data to AoRRP alone, we noted a trend for men to have more aggressive disease as compared to women (37% vs 11%, odds ratio = 4.6, Fisher exact p = 0.063). We also noted an association with country; 37% of Americans with AoRRP were aggressive; but in Lithuania, it was 12%; and in South Africa, it was 14% (Fisher exact p = 0.029). As was the case for all 339 subjects, the other variables, such as education, income, latency, race, and ethnicity were not associated with aggressiveness in AoRRP.

### Notwithstanding associations, ability to predict clinical course is modest

**Fig 3:**
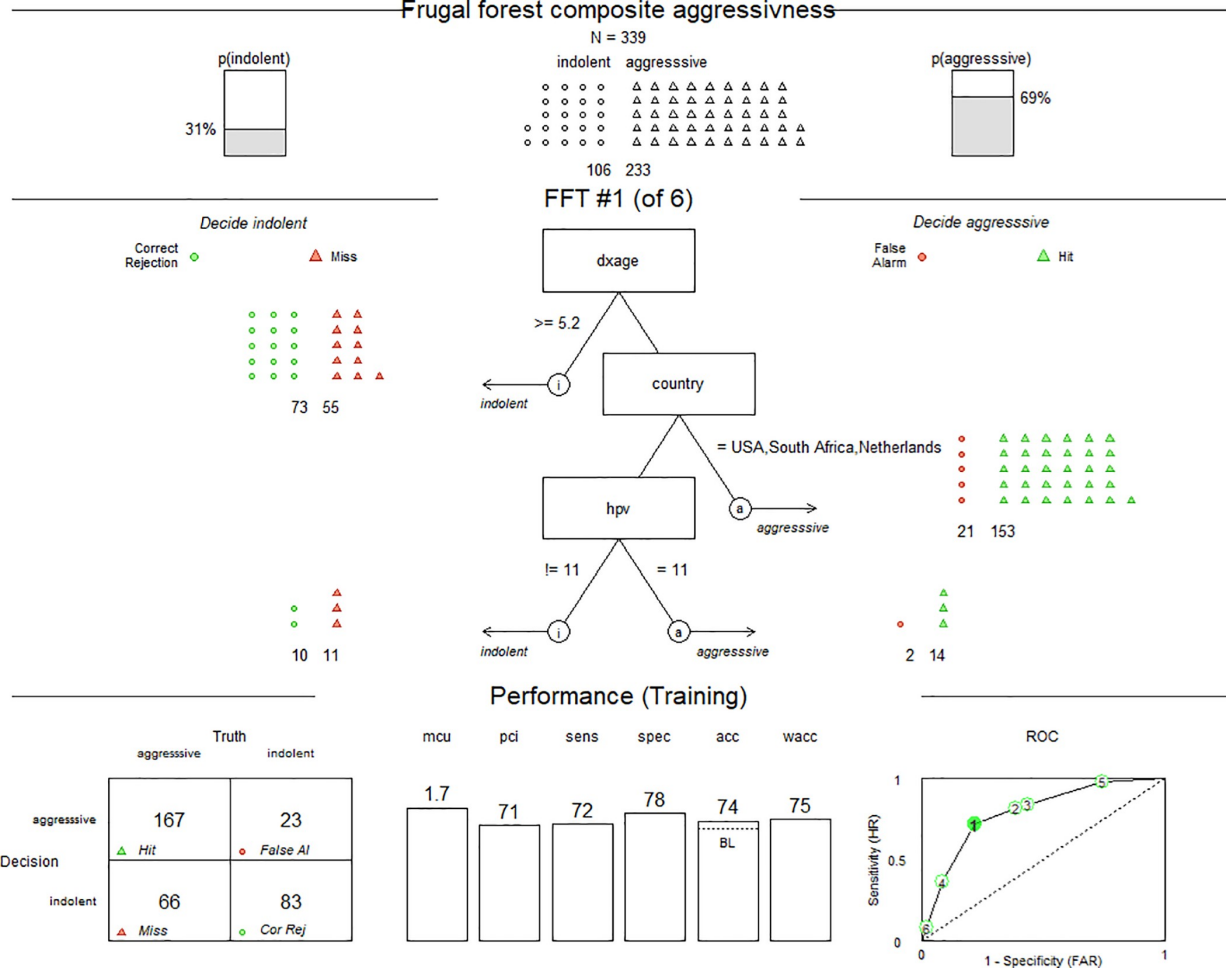
FFT for predicting the clinical course of RRP. A stepwise decision tree using age at diagnosis (dxage), country, and HPV type best predict the clinical course of RRP. An age at diagnosis of < 5.2 years, residence in the countries of USA, South Africa, and Netherlands, and HPV11 are the best variables to predict an aggressive clinical course of RRP. “p” is for probability. “! =“ is notation for “not equal to”. Colors: green = correct, red = false. Shapes: triangle = aggressive, circles = indolent (see bottom left 2x2 box).

A fast-and-frugal decision tree (FFT, Fig 3) was constructed to determine the best branches for predicting development of clinically aggressive disease [15]. The algorithm within the FFTrees package can determine which variables, the order thereof, and the best cutoffs to use and display the relative accuracy of competing trees. The top one-third of the FFT shows the total number of patients and a small chart displays the two groups. The probability that a random subject in this study has an aggressive clinical course is 69%. Since 233 of 339 subjects were deemed aggressive, the baseline prediction model would be to predict that every subject would have an aggressive course, a prediction that would be accurate 69% of the time, to the extent that this group is generalizable. The middle one-third panel (from top to bottom) of the FFT in Fig 3 demonstrates the best algorithm that can be generated from all the clinical metrics on file for all patients. The bottom panel presents the statistics behind the algorithm. The square chart at the bottom left shows the end result of patient classification if the algorithm were followed. The middle chart in the bottom one-third panel shows the mcu (mean cues used) of 1.7, pci (percent cues ignored) of 71, sensitivity of 72%, specificity of 78%, accuracy of 74%, and weighted accuracy of 75%. The plot in the bottom right panel shows a Receiver Operating Characteristic (ROC) curve. It shows six different outputs that the FFT could have used and the corresponding sensitivity and specificity trade-off of each. Plot 1 is bolded in the ROC curve to depict this algorithm in the middle one-third to be the most accurate of all the algorithms it came up with. The accuracy in this sample is 74% which seems of only limited utility when the baseline (BL) is 69%.

### Length of most recent surgical interval is associated with age and weakly with HPV type

A Welch two sample t-test utilizing log-transformed values was performed due to the significant skewness seen in the data. It demonstrates a significant difference in the days since last surgery between HPV6 versus HPV11. Patients with HPV6 had a geometric mean of 166 days since last surgery while patients with HPV11 had 98 days (p = 0.0014). A univariate linear regression model showed that the best predictor of variability in surgical interval was the age of the subject at the time of enrollment. Age accounted for 33% of the variability, whereas HPV accounted for only 3%, and country accounted for 20%. Age at diagnosis [18] and time since diagnosis [19] have both been shown to be associated with number of surgeries in the current year. We noticed that a model including both age at diagnosis and time since diagnosis explains more variability than simply age at enrollment (adjusted R^2^ 40% vs 32%). In subsequent model building, age at diagnosis plus time since diagnosis replaced age of enrollment. By adding country to the model, we were able to further account for the variability in the number of days between the last and second to last surgery (adjusted R^2^ from 40% increased to 44%). Compared to the USA, Lithuania was associated with more days between last and second to last papilloma debridement (p < 0.001), while in South Africa there was a trend to fewer days (p = 0.099). Adding HPV to the model, we see that HPV11 is associated with a shorter last intersurgical interval (p = 0.003), but the adjusted R^2^ increased minimally from 44 to 46%. None of the following variables were associated with the surgical interval: gender, race, ethnicity, other RRP in family, education, gross household income, maternal pap smear, cesarean section, maternal condyloma, paternal condyloma, latency, age of mother at time of birth, and time from maternal sexual debut to birth of affected subject.

Limiting the data to AoRRP alone, we also saw that increased time since diagnosis was associated with increasing last intersurgical interval. Age at diagnosis, unlike in JoRRP, was not associated with most recent intersurgical interval. There was a weak trend of men having a shorter intersurgical interval, but after controlling for the far stronger, and statistically significant, variables of country and time since diagnosis with multiple linear regression, there was no association with gender.

## Discussion

We report the largest international cohort of RRP patients that includes the entire spectrum of age at onset (both JoRRP and AoRRP) and for which HPV type data are available. Our analysis recapitulates many widely-held associations and provides new insights and confirmations. We noted three age-at-diagnosis brackets that are strongly associated with their own probability of an aggressive composite clinical course: below age 5, 5 up to 10 years, 10 and older. We also noted the weak and complicated relationship between HPV type and clinical course.

What is the purpose of collecting the data and then finding the best model that fits the data? There are two distinct audiences and/or purposes. Firstly, there is the purely academic mission of determining the scientific truth of the factors that cause RRP and that cause the most aggressive course and complications. Herein, our data set is sufficiently large that we can not only identify associated factors, but we also can determine the size of each factor’s effect, and the effect of any given factor while controlling for other associated factors. Such associated factors, if they are causative, could serve as targets of rational therapy. Secondly, there is the predictive mission. All that is being asked is to provide a prognosis so that doctors and affected individuals can anticipate the mostly likely clinical course. Almost always, variability of individuals is greater than variability of groups. With large datasets, it is quite common to find statistically significant differences between groups. However, it takes large effects and relative homogeneity within those groups for such a difference to provide useful predictions over and above the baseline probability of what happens to all affected individuals. Notwithstanding the effects of various factors that our models demonstrated, the models themselves predicted the clinical courses only slightly better than baseline predictions.

Our own group and several others have shown that in JoRRP, younger children are disproportionately infected with HPV11 and disproportionately follow an aggressive clinical course. Our publication in 2008 was the first to show that, after accounting for age at diagnosis, HPV type was not associated or only weakly associated with disease course [3]. Thereafter, several groups reported similar results [4,5]. Omland et al, analyzing Norwegian patients, noted that HPV type was not associated with clinical course in those with JoRRP when controlling for age. However, when age had no association, as in AoRRP, HPV11 was indeed associated with an aggressive course, but only when controlling for length of follow up. In a univariate model, HPV11 was only slightly associated with aggressiveness (odds ratio 1.2, p = 0.28). Tjon Pian Gi et al were the first to demonstrate that HPV11 had a contrasting association with aggressiveness in adults vs children [20]. In a single multiple logistic regression model, we have demonstrated an interaction between HPV type and age at diagnosis: in JoRRP, HPV11 is associated with aggressive disease, whereas in AoRRP, HPV11 is associated with indolent disease. That contrast is statistically significant. Across all patients with RRP, however, HPV11 is not associated with a greater proportion of aggressive clinical course, when controlling for age at diagnosis.

In addition to the composite (see definition in methods), intersurgical interval serves as yet another metric to quantify disease aggressiveness. It does not suffer from some of the weakness of composite endpoints. Composites are driven by the most common qualifier (i.e. total number of surgeries > 10) at the expense of the least common qualifier (i.e. tracheotomy). Further, composites suffer from being right censored; a subject who was only diagnosed 1.5 years before enrollment might not yet have had an opportunity to accumulate 10 lifetime surgeries but may be on their way there. A subject whose clinical course is currently labelled “indolent” and who has had 6 debridement interventions, might stay “indolent” if they had only a couple more interventions before going into remission, or the subject could transition to the label “aggressive” if there was 5 more interventions over the subsequent 2 years. In turn, surgical interval has been criticized as a metric of aggressiveness since so many factors can influence the interval and it can be variable [21]. Notwithstanding, the most recent intersurgical interval is a cross-sectional snapshot that demonstrated very similar associations as composite aggressiveness. Once again, age at diagnosis far more than HPV type was associated with the most recent intersurgical interval. Even more than age at diagnosis, we see that the greater the time since diagnosis, the longer the number of days since the last surgery. For a plurality of patients, as time goes on, their need for debridement decreases [19].

RRP in a particular individual has several attributes that could differentiate severe from mild disease at a particular point in time: size of papilloma, anatomic extent of disease, time since last debridement procedure (often referred to as intersurgical interval), presence of stridor, urgency of current intervention etc. Those moment-in-time metrics may be used as is and compared to paired bioassays in specimens taken at the same time. For example, one could measure mRNA expression levels in a papilloma biopsy taken at that same point in time or perhaps pair the metrics with peripheral blood mononuclear lymphocyte proliferation tests.

It is common for researchers to aggregate serial moment-in-time metrics. For example, Abramson et al quantified disease severity by documenting the number of disease sites, the surface area involved and the extent of luminal obstruction at each and every direct examination of the airway [22]. The scores were divided by the intersurgical interval yielding a growth rate. In 1998 the Derkay-Coltrera staging system was proposed by the authors of the widely used severity scales at the time [23]. The new system added functional measures and the article included a critique of several earlier scoring systems. The intention was to stage patients during each airway intervention and widespread adoption was encouraged so that results of interventions by various researchers could be compared.

For some analyses, a snapshot in time or a before-vs-after scoring/staging could be illogical. For instance, if observing age at diagnosis or HPV type or germline mutation, we would prefer to determine the association with the disease course as a whole. We would want to aggregate all the snapshots and summarize the clinical course with a single or a perhaps several metrics. If one had high resolution clinical data, one could choose an approach analogous to differential calculus, where one calculated the maximum or minimum of any metric (eg maximum anatomic extent, minimum intersurgical interval). One could also use an approach analogous to integral calculus, where one calculated the area under the curve (eg number of papilloma x size of papilloma x duration) or one could use composite outcome metrics as we have used here. The current study was initially conceived as a massively multi-centered, low budget (funded by a National Institutes of Health Mentored Clinical Scientist Development Award (K08)), germline genetic susceptibility study. We pondered the tradeoff between high resolution data over the entire disease course versus the burden placed on each collaborator. Our purpose was best met by using the widely cited composite aggressiveness scale of Doyle et al. We do not have access to the high resolution data that constitute the other measures of disease course.

From the earliest studies, it has been known that JoRRP is associated with a more aggressive course than AoRRP; and even within JoRRP, onset at younger age has been associated with more aggressive course than older age. Our large dataset permitted exploratory data analysis in which we could visualize the proportion of subjects who went on to have an aggressive clinical course as a function of age at diagnosis. It appears that the proportion remains high to the age of 5 years, drops between the ages of 5 and 10 years, and remains low thereafter.

The reason for the relationship between young age and aggressiveness is not known but may reflect airway size and/or the dynamics of the neonatal and infant immune system. Most people who encounter HPV, clear [24], or at least contain, the virus and the quintessential question in all HPV disease is why and how do the unfortunate few develop pathology. Across the spectrum of HPV diseases, evasion of the immune system, through multiple pathways, has been demonstrated [25]. In RRP, the exploration of a HPV-specific immunodeficiency has been led by Vincent Bonagura and his collaborators and mentees at North Shore-LIJ Health System, NY, USA. Over several decades and many studies, they have shown a recurring theme of a down regulated innate and adaptive immune response to HPV6 and HPV11 in those with RRP and even more so in those with the most severe disease. The downregulation is most evident within the papilloma itself. Papillomas resolve when large numbers of lymphocytes infiltrate the neoplasm [26] but alas in RRP, that either does not happen or is slow to occur. HPV-specific T cells from RRP patients showed reduced IL-2 secretion and behave as if anergic [27]. T_h_2-like chemokines are overexpressed in the plasma of patients with RRP and correlate with disease severity [28]. Further, these chemokines are produced by infected keratinocytes thereby inhibiting a local adaptive immune response. Plasma levels of the same chemokines decrease as patients go into remission. Even at the level of germ line genetic susceptibility, there have been associations found between the major histocompatibility complex and RRP. Bonagura et al demonstrated that HLA DRB1*0301 and DQB1*0201 are disproportionately present in people with RRP [29] and may influence the disease course. Notwithstanding these immunologic insights, there is no RRP-specific clear line from these mechanisms of HPV-specific anergy through the immunologic dynamics in the first five years of life. Or perhaps it has less to do with the age at diagnosis being less than 5 years and more to do with incident infection having taken place at birth. The neonatal innate immune system is biased against the production of pro-inflammatory cytokines (strong T_h_2-cell bias) [30] and predisposed towards tolerance. The chronology of incident infection has been studied in women. The humoral immune response is slow, weak and many do not seroconvert [31]. Perhaps that immune system is more likely to be absent or be slower in neonates than in older children and adults.

The significance of country in modeling clinical course parameters is at once fascinating, novel, curious and doubtful. Perhaps the data reflect access to healthcare, which varies from country to country. Maybe climate influences disease course. Perhaps population stratification and genetic variance from country to country result in variation in susceptibility to aggressive disease. It is possible that immunological function varies as a result of variations in nutritional status or in HIV status between countries. It might be that the circulating HPV types and lineages influenced the disease course. We do know that the proportion of HPV types differ from country to country (Fisher exact test p = 0.014). HPV6 accounted for 65% of the HPV types isolated in the whole cohort yet the following countries’ observed counts demonstrated the most statistical deviation from the expected count: South Africa (45% HPV6, 23/51), USA (69%, 148/213) and Colombia (33%, 2/6)). Notwithstanding these differences, HPV type had little association with the outcome. However, the association with country could be spurious since we used a convenience cohort and made no attempt to obtain a random sample of all RRP cases from each country. Furthermore, while in this data set, the USA was represented by 22 institutions, the other 7 countries were represented by 1 or 2 institutions. It is quite possible that instead of seeing a country to country variation we are simply seeing a city to city variation or an institution to institution variation or even a doctor to doctor variation. Expressed statistically, surgeons and institutions are nested within countries and thus are not amenable to disentanglement.

Over the years, research groups have wondered about socioeconomic associations with RRP. Our data do not permit any exploration of associations with incidence, but they do permit explorations with disease course. Despite asking about several indicators (e.g. education level, gross household income etc.) we found no association between these variables (either alone or when controlling for country) and aggressive vs indolent course. Markers of maternal HPV disease have been associated with RRP. Silverberg et al demonstrated a greatly elevated relative risk of developing RRP among those Danish children whose mother had a history of genital warts [32]. Perhaps the maternal condyloma serve as a proxy for HPV6 or 11 infection of the genital tract. Even more interesting are findings that show that maternal manifestations of HPV infection are not just associated with increased incidence but with a more aggressive clinical course as shown in Denmark [1] and in Germany [33]. We found a weak trend in the same direction.

The data reported herein have several limitations. The subjects represent a biased sample that is likely over-represented for aggressive RRP cases. In addition, the subject acquisition strategy was initially developed to study underlying genetic susceptibility to RRP. Nevertheless, the data set is an invaluable resource for exploring internal contrasts such as HPV6 vs 11 differences, since neither the patient nor the attending surgeon usually had knowledge of viral type during the management of the patient and during data acquisition. The 339 subjects that make this dataset are most certainly not a random sample of the population of people with RRP. It is a convenience sample heavily enriched with patients whose attending otolaryngologist has a special interest and dedication to the academic pursuit of understanding RRP. We reasonably expect patients with an aggressive and long course to be disproportionately represented. Hundreds of surgeons were invited; the efforts of 50 collaborators in 33 institutions generated this data set. The study did not keep screening logs, and we do not know the frequency or characteristics of those patients who were never invited or who declined. We believe that those following a protracted, aggressive course would be more likely to be invited and be more likely to join a research study than those with an indolent course. Repeated measures and ongoing longitudinal follow up was never built into the data acquisition. We asked about location of papillomas in the airway but not by subsite within structures; within the larynx, we did not record if the anterior commissure was involved or not. We did not ask about malignant transformation. Remission and its timing may be one of the most variable and hoped-for events in a clinical course, but only a dedicated research registry designed to quantitate such events would include contact with patients long after papilloma had stopped growing. Recurrence after long remissions have been observed but have not been studied systematically and we do not know their frequency and timing.

A finding that age at diagnosis under 5 years is associated with worse outcomes and the lack of an association between age and/or latency in AoRRP leads us to believe that young diagnosis in a child is probably the cause of a worse course and not merely a manifestation of it. In both adult and juvenile onset, the time between surgeries, in aggregate, increases as a patient lives longer and longer with disease. HPV type either has no statistically significant association with outcomes of composite aggressiveness or if it does then the association is weak in contrast to age at diagnosis. Composite aggressiveness classification is driven mostly by the number of surgeries and the most frequent number in a 1-year period but there are less common and more wretched components of the composite. The uncommon and devastating phenomena of pulmonary RRP and tracheotomy are being explored in a separate paper.

In JoRRP, the greatest association with aggressive disease is with age at diagnosis, but in AoRRP there is no such association other than the baseline fact that the majority (70%) run an indolent course. If there is any HPV association, then in young children it is with more aggressive disease; and this is certainly not the case in AoRRP where the association has a trend in the opposite direction. Both in JoRRP and AoRRP, there is an association between HPV11 and younger age at diagnosis, but in AoRRP there is no association between HPV type and latency.

In conclusion, even after an exhaustive analysis of a large data set, our ability to predict the outcomes in RRP patients is limited and only slightly better than using baseline probabilities. Time-based metrics (e.g. age) explain most of the clinical course variability within our sample. Perhaps immune maturation studies, host genetics or HPV genetics will provide additional insights. Future research should include high resolution long term follow up. On the one hand, patients who already have RRP seek remission; on the other hand, children born to mothers with HPV6 or 11 need an effective strategy to prevent RRP altogether.
